# Laparoscopic reduction of acute intrathoracic herniation of colon, omentum and gastric volvulus

**DOI:** 10.4103/0972-9941.26650

**Published:** 2006-06

**Authors:** Vishwanath Golash

**Affiliations:** Department of Surgery, Sultan Qaboos Hospital, Salalah - 211, Sultanate of Oman

**Keywords:** Gastric volvulus, herniation of colon and omentum, laparoscopy

## Abstract

Intrathoracic gastric volvulus with herniation of colon and omentum in a paraesophageal hernia is a rare occurrence. It may present as an acute surgical emergency with life-threatening complications. The diagnosis is usually made by imaging studies and endoscopy. Definite treatment is surgery. We present the laparoscopic management of this case.

## INTRODUCTION

Herniation of stomach and other viscera has been reported in type IV paraesophageal hernia, leading to serious complications such as perforation of the viscera, bleeding and gastric volvulus. The laxity of the supportive ligaments and diaphragmatic defect predisposes to gastric rotation and also the herniation of viscera. The herniation of viscera and gastric volvulus may be asymptomatic or present with chronic intermittent reducible hernia progressing to acute obstruction, as happened in this patient. The patient′s symptoms depend on the degree of obstruction and twisting. Clinically upper abdominal pain, retching and inability to pass a nasogastric tube would lead to clinical suspicion of acute gastric volvulus, but when associated with herniation of colon and omentum, the picture may be more confusing.[1,2] X-ray chest, endoscopy, CT scan, MRI scan, barium studies, all contribute to the diagnosis.

## CASE REPORT

**Figure 1:**
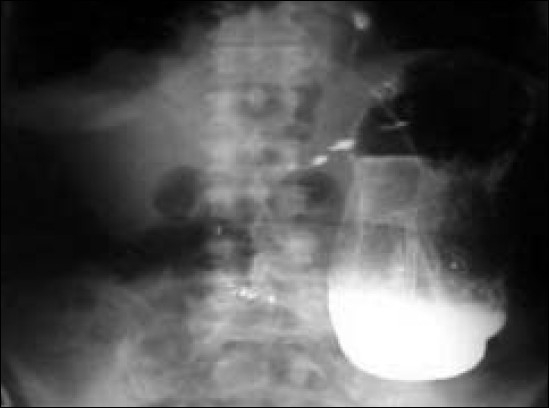
Barium meal after successful reduction of intrathoracic gastric volvulus. A hugely dilated stomach reaching to the pelvis with beaked pylorus suggesting outlet obstruction secondary to organoaxial torsion. Colonic loops were seen entering the chest which were not seen previously prior to the reduction of stomach

A 75 years old man who was previously asymptomatic, presented to us with, history of severe epigastric and chest pain, inability to eat, vomiting and acute constipation. His symptoms were getting gradually worse for last four days. On examination, he was moderately dehydrated and his vital signs were stable. Apart from upper abdominal tenderness, he had no other findings per abdomen. His investigations revealed hypokalemia, hyponatremia and hypoalbuminemia. The blood gases, serum amylase, ECG and troponin were normal. Chest X-ray showed a large air fluid level. After adequate resuscitation, a gastroscopy was arranged, which showed severe esophagitis, gastroesophageal reflux, a large hiatus hernia with the stomach in the chest and huge dilatation of stomach. The stomach was aspirated and a nasogastirc tube was inserted. An urgent CT scan of chest and abdomen with contrast, confirmed the intrathoracic volvulus of the stomach. The omentum was displacing the lower esophagus in the chest. Subsequently, a barium meal was arranged to define the exact anatomical problem. It confirmed the findings of endoscopy and showed an upside down stomach in the chest. The stomach was decompressed, draining out over two liters of fluid. This relieved the pain and discomfort in his upper abdomen and chest. Follow-up films showed complete reduction of stomach in the abdomen and a dilated stomach reaching to the pelvis with beaked pylorus, suggesting outlet obstruction secondary to organoaxial torsion. The colonic loops were seen entering the chest, which was not seen previously prior to the reduction of stomach. Late films showed the partially contrast-filled right colon entering the chest [Figure 1].

**Figure 2:**
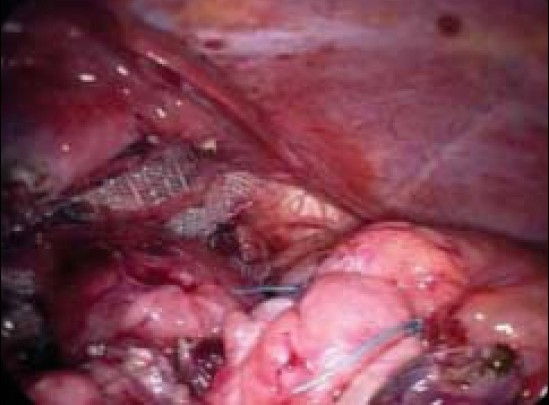
The mesh reinforcement of hiatus and fundoplication

The patient was placed supine in the 30° anti-Trendelenburg position. A 10 port was inserted a hands breadth below the xiphisternum for the 0° laparoscope and further five mm ports were inserted along the costal margin in the mid line, right and left midclavicular line and the left anterior axillary line. The diagnosis was confirmed at laparoscopy. The stomach had herniated again in the hiatus and only pylorus ends were intra-abdominal. The greater omentum and transverse colon were seen entering the hiatus. The greater omentum was easily reduced by hand-over-hand technique, followed by colon and stomach. The hernial sac was excised and cruroplasty was done by interrupted intracorporeal non-absorbable sutures. There was a huge hiatal defect left after the cruroplasty, which was bridged by a circular 10 × 10 cm. Polypropylene mesh.[3] A Nissen fundoplication and anterior Gastropexy was done to prevent the reflux and recurrence [Figure 2]. The abdominal cavity was drained.

His repeat barium studies, six months after surgery was normal.

## DISCUSSION

The decompression of the stomach was successful in this patient in reducing the gastric volvulus, which was confirmed by subsequent chest X-ray and follow up barium meal. But on laparoscopy, the stomach was seen to be rotated again on its long axis and had herniated in the chest. We think that this represents a sequential recurrent acute gastric volvulus in a para esophageal hiatus hernia. Most likely, explanation of this patient becoming symptomatic and progressing to acute obstruction, may be due to slow expansion of his chronic gastric volvulus, as well as the incarceration of colon and omentum or the combination of all. Surgery is recommended at the time of diagnosis because of the risk of strangulation and high mortality. A definitive treatment is always surgical. A laparoscopic approach has the advantage, as it minimizes the trauma and identifies the underlying predisposing conditions. The various laparoscopic therapeutic strategies adopted include: reduction, excision of sac, repair of hiatus with or without prosthesis, fundoplication, laparoscopic anterior gastropexy, laparoscopic gastrostomy fixation, laparo-endoscopic reduction and percutaneous gastrostomy, laparoscopic esophagocardiopexy and gastrectomy. All these modalities have been used alone or in combinations depending on case to case basis.

The reduction may be successful after decompression through the use of nasogastric tube or endoscopy like in this case and should always be attempted; an elective laparoscopic repair can be performed later to allow time for stabilization of the patient condition. Failure of nasogastric and endoscopic decompression requires urgent surgery.

A laparoscopic reduction and anterior gastropexy alone may be a satisfactory answer in an emergency situation.[4] A fundoplication can be performed later. Rarely, has herniation around an anterior gastropexy requiring second surgery to repair the defect, been reported.

A fundoplication is recommended to secure the stomach in the abdomen and to prevent the existing or possible postoperative gastro-esophageal reflux. A temporary gastrostomy may be added in addition to hernia repair, to fix the stomach to anterior abdominal to prevent the recurrence. The location of gastrostomy tube is important, as recurrence of volvulus has been reported after fundoplication and gastrostomy, acting as two fixed points of the axis.

The endoscopic decompression with percutaneous endoscopic gastrostomy or laparoscopic derotation and laparoscopic gastrostomy, has been shown to give satisfactory results in elderly medically unfit patients.[5]

Repair of hiatus is necessary to reduce the recurrence of volvulus. Prosthetic reinforcement of the hiatus hernia defect which cannot be closed primarily by cruroplasty, is safe and effective and should be the option in the treatment of a large hiatus hernia with intrathoracic stomach and colon. Several kinds of prosthesis are in use, the difference being in their foreign body reactivity and hence in their adhesive potential. Although in use for several years, the prolene polypropylene meshes are known to give rise to adhesions. Rarely has erosion into the esophagus, also been reported. The newer modifications of polypropylene meshes are claimed to be helpful in preventing these complications.[3]
